# Extracellular Vesicle-Based Therapy for COVID-19: Promises, Challenges and Future Prospects

**DOI:** 10.3390/biomedicines9101373

**Published:** 2021-10-01

**Authors:** Vamika Karn, Shaista Ahmed, Lung-Wen Tsai, Rajni Dubey, Shreesh Ojha, Himanshu Naryan Singh, Mukesh Kumar, Piyush Kumar Gupta, Soumi Sadhu, Niraj Kumar Jha, Ashutosh Kumar, Soumya Pandit, Sanjay Kumar

**Affiliations:** 1 Department of Biotechnology, Amity University, Mumbai 410221, India; vamika398@gmail.com; 2 Faculty of Medical and Paramedical Sciences, Aix-Marseille University, 13005 Marseille, France; shaista.ahmed@etu.univ-amu.fr; 3 Department of Medicine Research, Taipei Medical University Hospital, Taipei 11031, Taiwan; lungwen@tmu.edu.tw (L.-W.T.); 205095@h.tmu.edu.tw (R.D.); 4 Department of Information Technology Office, Taipei Medical University Hospital, Taipei 11031, Taiwan; 5 Department of Pharmacology and Therapeutics, College of Medicine and Health Sciences, UAE University, Al Ain, Abu Dhabi P.O. Box 17666, United Arab Emirates; shreeshojha@uaeu.ac.ae; 6 Department of System Biology, Columbia University Irving Medical Center, New York, NY 10032, USA; hs3290@columbia.edu; 7 Department of Biophysics, All India Institute of Medical Sciences, New Delhi 110029, India; krmukesh11@gmail.com; 8 Department of Life Sciences, School of Basic Sciences and Research, Sharda University, Greater Noida 201310, India; piyush.kumar1@sharda.ac.in (P.K.G.); soumi.sadhu@sharda.ac.in (S.S.); soumya.pandit@sharda.ac.in (S.P.); 9 Department of Biotechnology, School of Engineering & Technology (SET), Sharda University, Greater Noida 201310, India; niraj.jha@sharda.ac.in; 10 Department of Anatomy, All India Institute of Medical Sciences, Patna 801507, India; drashutoshkumar@aiimspatna.org

**Keywords:** COVID-19, extracellular vesicles, SARS-CoV2, therapeutic agents

## Abstract

The COVID-19 pandemic has become a serious concern and has negatively impacted public health and the economy. It primarily targets the lungs, causing acute respiratory distress syndrome (ARDS); however, it may also lead to multiple organ failure (MOF) and enhanced mortality rates. Hence, there is an urgent need to develop potential effective therapeutic strategies for COVID-19 patients. Extracellular vesicles (EVs) are released from various types of cells that participate in intercellular communication to maintain physiological and pathological processes. EVs derived from various cellular origins have revealed suppressive effects on the cytokine storm during systemic hyper-inflammatory states of severe COVID-19, leading to enhanced alveolar fluid clearance, promoted epithelial and endothelial recovery, and cell proliferation. Being the smallest subclass of EVs, exosomes offer striking characteristics such as cell targeting, being nano-carriers for drug delivery, high biocompatibility, safety, and low-immunogenicity, thus rendering them a potential cell-free therapeutic candidate against the pathogeneses of various diseases. Due to these properties, numerous studies and clinical trials have been performed to assess their safety and therapeutic efficacy against COVID-19. Hence, in this review, we have comprehensively described current updates on progress and challenges for EVs as a potential therapeutic agent for the management of COVID-19.

## 1. Introduction

In December 2019, an outbreak of pneumonia fever was reported in Wuhan, Hubei Province, China with an unknown cause of infection. Later, in January 2020, a novel coronavirus was isolated from infected patients, which was termed SARS-CoV-2 (severe acute respiratory syndrome coronavirus 2) [1,2,3,4,5]. Owing to its occurrence in 2019, the World Health Organization (WHO) designated this infection as coronavirus disease 2019 (COVID-19), which has created an alarming situation through higher global mortality rates. Before COVID-19, the world had witnessed two similar strains of coronaviruses in the early 21st century, which were the severe acute respiratory distress syndrome coronavirus (SARS-CoV) and the Middle East respiratory syndrome (MERS-CoV) [6].

**Figure 1:**
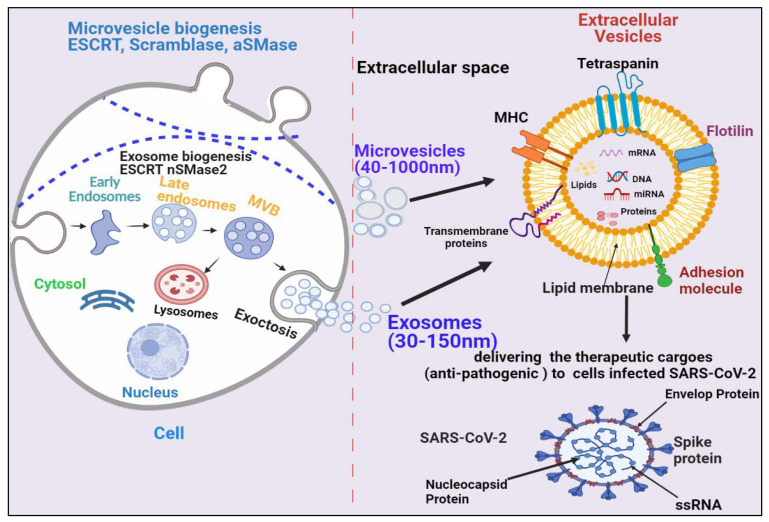
Biogenesis and secretion of EVs (microvesicles and exosomes) and their therapeutic role in COVID-19. The secretion of exosomes into the extracellular environmentundergoes three distinct steps: exosome biogenesis, intracellular trafficking of MVBs, and fusion of MVBs with the plasma membrane. Microvesicles are synthesized through direct outward budding and detachment of the plasma membrane into the extracellular milieu. Several molecules are involved in the biogenesis of both microvesicles and exosomes (small GTPases, ESCRTs, ARRDC1, syndecan, ceramide, tetraspanins). These EVs binds to SARS-COV-2-infected cells and deliver their therapeutic cargos to inhibit their pathogenesis.

The SARS-CoV-2 virus comes under the family Coronaviridae and order of Nidovirales. The genome of SARS-CoV-2 is single-stranded, positive sense 26–32 kb large RNA with nucleocapsid (N) protein, which is packed within an envelope and responsible for viral replication in host cells. The virus envelope is made up of three types of structural proteins i.e., membrane (M), spike (S), and envelope (E) proteins. Among these, trimeric spike (S) glycoproteins protrude from the envelope (Figure 1) and play a key role in virus entry into host cells [7].

Clinical manifestations, such as acute respiratory distress syndrome (ARDS) and some immune-mediated lung complications have been associated with poor prognosis of COVID-19, which lead to multiple organ failure such as heart, liver, kidney, and brain—primarily in elderly patients, but also later in young individuals, causing an increased death rate [8]. Ideally, an incubation period of coronavirus is about 5 days, but may also range from 2 to 14 days [9]. Following the initial symptoms of COVID-19, hypoxemia and pneumonia fever progress, leading to the requirement of a ventilator support system [7,8,9]. The possible root cause of the higher mortality rate of COVID-19 patients is hypoxemia and respiratory failure, that lead to lung injury with several other complications like edema, intra-alveolar fibrin deposition, and hemorrhage resulting in ARDS [9]. It has been observed also that individuals with a history of cardiovascular disease, lung disorder, hypertension, and diabetes are at higher risk of COVID-19 infection [10].

Given the present circumstances around the world, to date, various potential drugs are being clinically tested against COVID-19; however, their adequate efficacy remains to be achieved. Therefore, it is important to develop an alternative therapeutic strategy for infected patients and to stop the chain of SARS-CoV-2 transmission. In recent years, EVs have shown promising anti-inflammatory properties against viral infection [11]. EVs are lipid bilayer membrane-bound structures, which are released from various kinds of cells, and contain many bioactive compounds (cargo) such as mRNAs, microRNAs (miRNAs), DNA, lipids, and various proteins. Due to their intact structure, EVs can circulate through body fluids freely and can deliver their cargo to neighboring or remote cells to help maintain their physiological condition [12].

Based on their size and biogenesis, EVs have been categorized into three types, which include exosomes, microvesicles, and apoptotic bodies. Exosomes are smaller in size (30–120 nm) and synthesized by the endosomal pathway, involving the formation of intraluminal vesicles (ILVs) inside multivesicular bodies (MVBs) in the cytoplasmic compartment of cells. MVBs bind to the inner plasma membrane, releasing their ILVs in the extracellular environment in the form of exosomes. There are several proteins involved in exosome biogenesis such as endosomal sorting complex required for transport (ESCRT), vacuolar ATPase, and Vps4, which segregate and sort ubiquitylated proteins into ILVs (Figure 1) [13,14,15,16,17,18]. Microvesicles are a little larger (40–1000 nm) in size and are released through pinching-off the plasma membrane via a direct budding process. Similar to exosomes, multiple protein factors also participate in microvesicle generation, such as Ca^2+^-dependent aminophospholipid translocases (flippases and floppases), sphingomyelinase 2 (nSMase2), scramblases, and calpain, which carry out the rearrangement of phospholipids, curving of the membrane, and reconstitution of the actin cytoskeleton, leading to pinching of the membrane in the form of microvesicles in extracellular milieu (Figure 1), [19,20,21,22]. Apoptotic bodies are the largest (greater than 1000 nm) and are synthesized during the apoptosis process.

EVs have been demonstrated to enhance lung immunity, and have been implicated in the pathogenesis of many types of lung diseases that include viral infection. This might be attributed to the structural similarity between SARS-CoV-2 and EVs (Figure 1) [11]. Recent studies have also shown that viruses employ EVs to exit from cells, while EVs use a virus penetration mechanism for cargo delivery [23]. Hence, EV-virus interactions could be utilized for the development of antiviral vaccines and drugs to terminate viral pathogenesis. Despite EVs’ role in viral pathogenesis, their therapeutic potential has also been explored in many studies, which are discussed in the following section.

## 2. COVID-19-Associated Multiple Organ Failure and EV-Mediated Recovery

**Figure 2:**
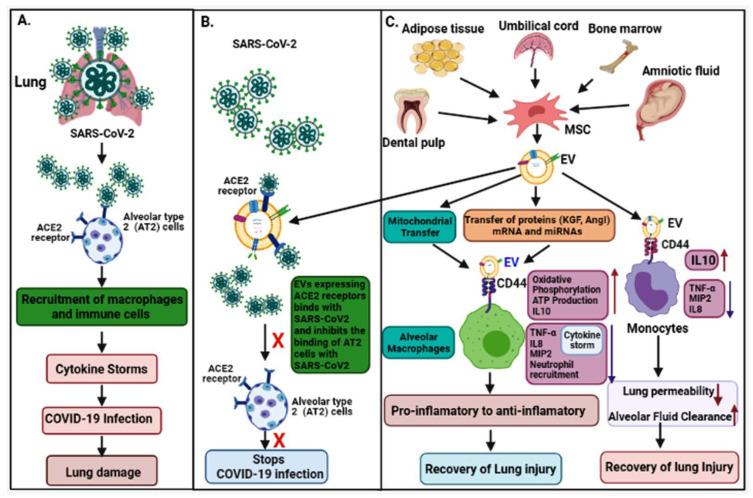
The pathogenesis of COVID-19 and its EV-mediated therapeutic recovery. (**A**) SARS-CoV-2 binds to ACE2 receptors on alveolar type 2 (AT2) cells in the lung and induces cytokine storms leading to lung damage. (**B**,**C**) Therapeutic effects; ACE2 receptors expressed on MSC-derived EVs competitively bind to SARS-CoV-2 and inhibit the binding of the virus to AT2 cells, and consequently inhibit the viral infection. MSC-derived EVs transfer mitochondria, proteins (KGF, and AgoI), mRNA and miRNAs via binding to CD44 receptors on macrophages, suppress the cytokine storm (IL-8, TNF-α, MIP2) and enhance anti-inflammatory cytokines (IL-10), ATP production, and oxidative phosphorylation—which helps in recovery from lung injury. MSC-derived EVs also bind to monocytes via CD44 receptors, repress the cytokine storm, and enhance anti-inflammatory cytokines IL-10, leading to recovery of the injury.

**Figure 3:**
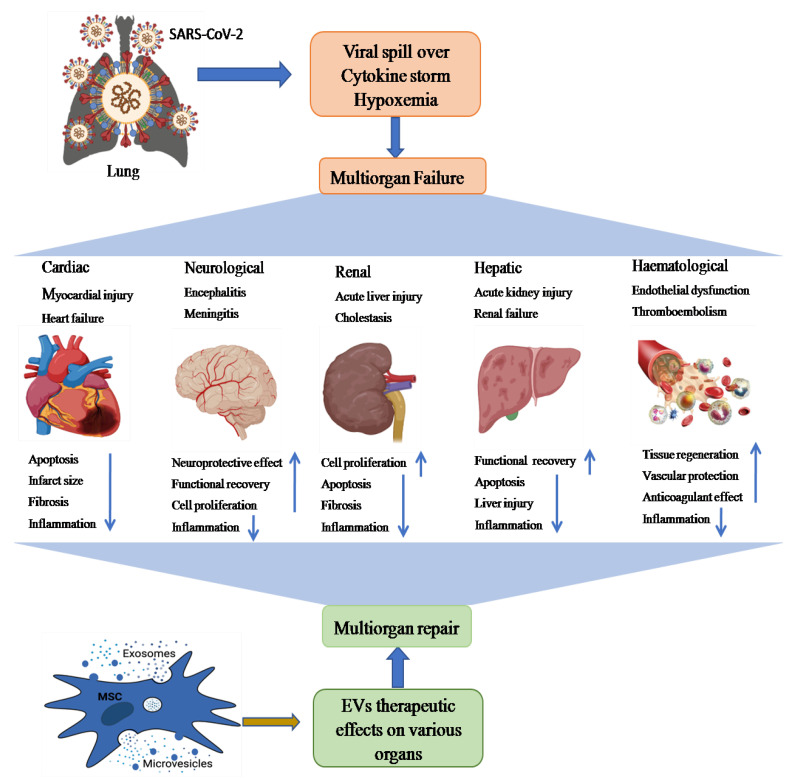
A schematic representation showing possible paths of MSC-EV (exosomes and microvesicles)-mediated therapy of SARS-CoV-2-induced multiple organ failure (heart, kidney, liver, brain injury, and hematological disorders). Multiple organ dysfunction mainly occurs by binding ACE2 receptors on different organs, cytokine storm, and hypoxemia. These multiorgan pathological aberrations could be recovered through anti-inflammatory, tissue regenerative, and neuroprotective effects of EVs.

Lungs are the first organ where SARS-CoV-2 virus enters and causes infection (Figure 2). In addition to the lungs, other vital organs such as the heart, kidney, liver, brain, and blood vessels are also infected in severely ill COVID-19 patients, which renders it a systemic disease (Figure 3). Although the mechanistic insight underlying multiple organ infection in COVID-19 is yet to be described, it might be mediated in two possible ways. Firstly, SARS-CoV-2 may enter directly into different organs by binding angiotensin-converting enzyme 2 (ACE-2) receptors, which are expressed on the cellular surfaces of the major human vital organs [24,25]. Secondly, SARS-CoV-2 infects the lungs and induces cytokine storms, infiltration of inflammatory cells to tissues, and coagulation dysfunction, which adversely impacts various organs and may lead to multi-organ damage [26]. Among multiple possible ways to tackle the COVID-19 pandemic, EVs have emerged as a potential cell-free therapy. Based on this, EV-mediated reparation and restoration of COVID-19-affected multiple organs is being investigated and has been highlighted in this section.

### 2.1. COVID-19-Associated Lung Damage and Its Recovery by EVs

The epithelium lining of lung alveoli comprises a single layer of alveolar type I (AT1) and type II (AT2) cells. AT1 and AT2 cells are firmly linked by tight junctions through which ions and fluids pass across the epithelium, whereas AT2 cells secrete a surfactant on epithelium linings to facilitate alveolar expansion. The expression of ACE2 receptors has been shown on lung surfaces, mainly on AT2 cells, along with resident alveolar macrophages [27]. SARS-CoV-2 binds ACE2 receptors expressed on target AT2 cells for their entry into the lung. Transmembrane serine protease 2 (TMPRSS2) expressed on alveolar cells is involved in priming of S (spike) protein of SARS-CoV-2 that enhances the infection of other alveolar cells [28]. This results in an elevated production of pro-inflammatory cytokines and chemokines that recruit more and more inflammatory macrophages and circulatory immune cells into the infected alveoli, which leads to a systemic over-inflammatory state called ‘cytokine storm’ [7,28]. Additionally, cytokine storms affect AT1 and AT2 cells that reduce the production of surfactants; this causes an increase in alveolar surface tension and collapse, as well as a decrease in gaseous exchange and refractory hypoxemia, and, ultimately, leading to ARDS [23,29]. 

Interestingly, higher levels of various cytokines such as IFN-γ (Th1), interleukins IL-1β, IL-2, 6, 7, 8, 17, monocyte chemoattractant protein-1 (MCP-1/CCL2), IFN-γ induced protein 10 (IP10), tumor necrosis factor-α (TNF-α), macrophage inflammatory protein-1α (MIP-1α/CCL3) and granulocyte-colony stimulating factor (G-CSF) have been reported in severe COVID-19 patients [2,30]. Pro-inflammatory cytokines produced by activated macrophages such as IL-1, IL-6, and TNF-α enter the bloodstream and increase the capillary permeability by dilating smooth muscle and contracting endothelial cells. Consequently, blood vessel plasma leaks into the interstitial spaces and causes alveolar edema [30]. 

It has been shown that EVs possess immunomodulatory effects that regulate macrophages by inhibiting TNFα secretion and enhancing anti-inflammatory IL-10 secretion [31]. Furthermore, MSC-derived EVs have been demonstrated to boost energy production by increasing mitochondrial performance in alveolar cells and increasing their repairing capability in the injured lung. Data has also suggested that human-derived EVs down regulate macrophage inflammatory protein-2 (MIP-2) levels and reduce lung inflammation by lowering the recruitment of neutrophils and preventing macrophage polarization into pro-inflammatory M1 macrophages [32,33]. Apart from MSC-EVs, neutrophil-derived EVs possess an anti-inflammatory role in lung epithelium via PARP-1 inhibition by miR-223 and enhance the recovery of the injured lung [34]. Recently, EVs have been shown to reduce lung edema and permeability of epithelial–endothelial barriers through binding with CD44 expressed on alveolar target cells [33]. Based on these findings, human MSC-exosomes might be a useful treatment approach in combating cytokine storms in COVID-19 patients.

### 2.2. COVID-19-Associated Cardiovascular Disease and Its Recovery by EVs 

The knowledge of COVID-19 impact on the heart is very crucial for healthcare providers to prescribe the appropriate treatment for patients. In a recent study, RNA-seq analysis revealed that ACE2 was expressed in over 7.5 percent of myocardial cells [35], suggesting that the heart might be at high risk of SARS-CoV-2 in case of viremia. In addition to myocardial cells, Gheblawi et al. also reported ACE2 expression in various other parts of the heart, mainly in cardiac fibroblasts, pericytes, epicardial adipose, and endothelial cells [36], thereby increasing the risk of direct infection by SARS-CoV-2 in heart tissue. An electron microscopy-based study showed particles consistent with COVID-19 virus present within a cardiac endothelial cell and also in CD4 and CD8-positive cells around the vascular endothelium, which suggests that immune cells can infiltrate to cardiac tissues [37]. Furthermore, histopathological examination of patients with COVID-19 reported a higher prevalence of fibrosis and myocyte hypertrophy in cardiac tissues [38], and hypothesized that cardiac tissue injuries may be caused indirectly by cytokine storms [39]. Additionally, coronary microvasculature dysfunction due to elevated cytokines levels can lead to myocardial injury [40]. In COVID-19 patients, Huang et al. demonstrated a high concentration of pro-inflammatory mediators such as IL-1β, IL-6, IL-12, monocyte chemoattractant protein-1 (MCP-1), IFNγ, and IFN-inducible protein, leading to coagulation activation [2,40]. In autopsy studies, megakaryocytes were detected in cardiac microvasculature and bone marrow, which suggested their having a role in diffusing microvascular thrombosis in COVID-19 patients [41]. These data suggest that COVID-19-related cardiovascular disease (CVD) may be induced either directly by SARS-CoV-2 infection in the cardiac system or indirectly via the virus’s cytokine storm, endothelial dysregulation, infiltrating immune cells, and microvascular thrombosis.

A significant proportion of COVID-19-infected individuals develop cardiac-related complications such as acute myocardial injury (AMI), arrhythmia, or heart failure [42], which necessitates the development of novel treatment strategies. Recently, EVs attracted great attention from researchers over the world because of their potential role in anti-inflammation, immunomodulation, and pro-angiogenesis [43]. Lai et al. (2010) first demonstrated the therapeutic potential of EVs—especially MSC-EVs—in the recovery of myocardial ischemia or reperfusion injury in a mouse model [44]. Various other in vivo studies also reported the protective role of MSCs-EVs in AMI [45,46]. Arslan et al. showed that a single dose of intravenous injection of MSC-derived exosomes resulted in reduced infarct size and oxidative stress, and enhanced NADH and ATP levels, which are a sign of recovery of reperfusion injury in the mouse AMI model [45]. Later, Bian et al. reported a potential pathway involving MSC-EVs in repairing ischemic myocardial injury by inducing neovascularization [47]. Several other notable effects of EV-mediated ischemic myocardial repair have been achieved by reducing fibrosis and apoptosis of myocardial cells [48,49]. One of these works in rats has shown that MSC-EVs from human umbilical cord participate in reducing cardiac fibrosis by preventing apoptosis of cardiomyocytes and enhancing cell proliferation [50]. These promising findings support our hypothesis regarding the therapeutic potential of MSC derived-EVs against COVID-19-related cardiovascular complications. 

### 2.3. COVID-19 Associated Kidney Diseases and Their Recovery by EVs

The kidney is one of the critical organs most severely impacted by COVID-19, which may manifest as damage in renal resident cells [41]. Recent reports have also confirmed that kidney disease is associated with the death of severely ill COVID-19 patients [51]. Infection caused due to virus–host cell interactions through ACE2 or the cytokine storm is assumed to be the underlying mechanism for renal injury [52]. Owing to ACE2 receptor-based virus–host cell crosstalk, ACE2 expression has been detected in various renal cells such as proximal tubule epithelial cells, glomerular endothelial cells, podocytes, and kidney vasculature [36]. A post mortem study of kidney biopsies from six COVID-19 patients with acute kidney injury (AKI) revealed macrophage and lymphocyte infiltration, as well as significant acute tubular necrosis. COVID-19 nucleocapsid protein (NP) antigen has also been found in kidney tubules and virus-like structures in the cytoplasm of renal tissue, tubular epithelium, and podocytes, suggesting that SARS-CoV-2 may infiltrate kidney cells directly [53]. Thus, a better understanding of the biology of kidney injury in association with COVID-19 is highly needed. 

It has recently been shown that EVs may play a role in the repair and regeneration of kidney tissue injuries by relaying signals between nephrons [54]. These signals may be delivered by EVs, which bind receptors and transfer cargo such as proteins, mRNAs, and miRNAs to their target cells [55]. Thus, the potential use of EVs as a therapeutic vector has gained significant attention in management of acute kidney injury [54,55]. Growing evidence has shown that MSC-derived EVs could reconstitute kidney structure and function in various in vivo models of acute kidney injury (AKI). Studies also suggest that MSC-EVs have been involved in immunomodulation and anti-apoptotic activities, thus enhancing cellular proliferation and protecting against renal damage [56,57]. In various animal models, MSC-EVs have been demonstrated to reduce pro-inflammatory cytokines and repair renal injuries [58]. Other studies also reported that activated macrophages infiltrate renal tissues and cause the progression of AKI. Thus, restricting infiltrating macrophages by EVs could be an important mechanism for recovery in AKI [59,60]. Later, Shen et al. discovered higher CCR2 expression on MSC-EVs, which could lower circulating CCL2 levels and reduce its ability to recruit or activate macrophages in renal tissues, and that CCR2 knockdown reduced the protective function of MSC-exosomes for renal I/R injuries in an in vivo model [61], indicating that receptor expression on EVs could play a key role in their therapeutic utility. Hence, more basic and clinical research is needed to have a better understanding of these pathways so that EVs can be used to treat COVID-19-related kidney damage and AKI.

### 2.4. COVID-19-Associated Liver Disease and Its Recovery by EVs

COVID-19 has been associated with acute liver injury (ALI), which is manifested by elevated levels of liver enzymes i.e., alanine aminotransferase (ALT) and aspartate aminotransferase (AST) [62]. Xu et al. demonstrated the pathological results of a COVID-19-related liver biopsy, which revealed moderate microvesicular steatosis and lobular activity, as well as portal inflammation [63]. Though the mechanism underlying this pathology is not fully understood, multiple theories have been proposed, which include direct ACE2-mediated injury in liver; specifically, the expression of ACE2 is very low in hepatocytes (2.6%), but cholangiocytes express 59.7% of total ACE2 in the liver, which is equivalent to their expression in AT2 cells, implying that the liver is another vulnerable target organ for SARS-CoV-2 [64]. Alternately, cytokine storm-mediated dysregulation of inflammatory and immune processes also contributes to hepatic fibrosis [65]. Besides pneumonia-related hypoxia, hypotension may also lead to liver damage or even failure in critically ill COVID-19 patients [66]. Reports have indicated that a variety of COVID-19 medications may also participate in hepatotoxicity that could contribute to liver damage [65,67]. However, these pathological outcomes have partially been treated by currently available therapeutic alternatives. Along with combating the virus, it is also essential to maintain the health of organs with suitable and targeted therapy. 

Recent advancements in pre-clinical studies have shown that MSC-derived EVs could exert positive impacts on liver diseases, such as liver fibrosis, inflammation, drug-induced liver injuries (DILI), and ALI in in vivo models [68]. Li et al., have shown that human umbilical cord MSC-derived-EVs could alleviate carbon tetrachloride-induced liver fibrosis in mice by inhibiting the epithelial–mesenchymal transition of hepatocytes and collagen synthesis [69]. Recently, engineered human umbilical cord perivascular cell (HUCPVC)-derived EVs have been shown to produce insulin-like growth factor-I (IGF-I) upon their administration, reducing hepatic fibrosis in mice [70]. Amnion MSC (AMSC)-derived EVs could reduce inflammation and fibrosis by downregulating the production of pro-inflammatory cytokines such as TNF-α, IL-1β, and IL-6,as well as inhibiting the expression of kuffer cells—particularly M1 macrophages in mice liver [71]. Additionally, embryonic MSC-derived EVs facilitate regeneration of hepatocytes in carbon tetrachloride-induced liver injury by activating the IL¬6/STAT3 pathway [68]. Furthermore, Lou et al. found that adipose tissue MSC-exosomes could reduce elevated serum ALT and AST levels as well as the production of pro-inflammatory cytokines in concanavalin A (Con A)-induced hepatitis in C57BL/6 mice [72]. Interestingly, liver stem cell-derived EVs have been shown to accelerate liver structural integrity and function in 70% of hepatectomized rats by promoting hepatocyte proliferation [73]. Based on these pieces of evidence, it could be inferred that EVs from different sources may prevent various types of liver disease by reducing inflammation and collagen production and enhancing hepatocyte proliferation. Since COVID-19-induced liver pathologies such as fibrosis, DILI, and ALI have already been reported, it is likely that MSC-derived EVs could be potential therapeutic candidates for such complications.

### 2.5. COVID-19 Associated Neurological Diseases and Their EV-Mediated Recovery 

Numerous studies have reported that COVID-19 is associated with several life-threatening neuropathologic manifestations such as encephalopathy, meningitis, and Guillain–Barre Syndrome [25,71,72,73,74]. In addition, the COVID-19 virus has been detected in human brain tissues and cerebrospinal spinal fluids (CSF) [75,76,77]. In the brain, ACE2 is expressed in neurons, astrocytes, and oligodendrocytes with higher prevalence in the motor cortex, posterior cingulate cortex, ventricles, circumventricular organs, thalamus, and olfactory bulb [76,78]. Furthermore, COVID-19 viral-like particles have also been detected in brain endothelial cells of autopsied patient tissues presenting at least one cell membrane bleb [75,79]. Although COVID-19 virus was not detected in primary human endothelial cells from brain tissues lacking ACE2 expression in vitro, endothelial cells over expressed with ACE2 were shown to promote infection in vivo [80], implying that COVID-19 infection in endothelial cells depends on the expression of ACE2. Currently, COVID-19 infected neurons have been associated with neurodegeneration and neurovascular alterations [81]. Elevated levels of inflammatory cytokines such as IL-6 and TNF-α have also been shown in the CSF of COVID-19 patients with neurological presentation, indicating an ongoing inflammatory process in the brain [82,83]. Cytokines such as IL-6, TNF-α, IL-1β, and IFN-γ, along with chemokines and acute phase C-reactive protein can disrupt and modulate the functions of the blood–brain barrier (BBB), which can influence adsorptive transcytosis [84,85,86,87]. Coagulation abnormalities due to high inflammatory responses leading to stroke were confirmed in COVID-19 patients [88]. Taken together, the above-mentioned evidence implies that COVID-19 infection may participate in damage, apoptosis, and dysfunction of brain microvascular endothelial cells and neurons, which may lead to neurological dysfunction [81,88,89]. Therefore, it is much needed to find a better therapeutic approach to facilitate positive clinical outcomes in COVID-19 patients.

Recent progress in EV research has demonstrated MSC-derived EVs as potential therapeutic tools for neurological disorders [90]. Specifically, exosomes may facilitate the functional restoration of neurological abnormalities by promoting neurogenesis and BBB integrity, suppressing inflammation and apoptosis, and leading to mitigated disease progression [91,92]. Bone marrow MSC-derived exosomes have been reported to suppress neuronal apoptosis and foster the functional recovery of the spinal cord after CNS injury by stimulating Wnt/β-catenin signaling [93,94]. Human umbilical cord MSC-derived exosomes can inhibit the activation of A1 astrocytes and act as anti-inflammatory mediators by regulating Nrf2/NF-κB signaling [95]. This study indicates that these exosomes may be a potential therapy for the treatment of inflammation-associated neurological dysfunction. In a preclinical cerebral hemorrhage stroke model, MSC-derived exosomes have been shown to support functional restoration and to remodel neurovascular defects [96,97]. In sum, these data show that EVs can traverse blood–tissue barriers to repair injured neurons during the development of COVID-19-related neurological disorders [90]. 

### 2.6. EV-Mediated Recovery of COVID-19-Associated Hematological Disorders 

Several hematological abnormalities like lymphopenia, thrombocytopenia, and coagulation defects have been associated with COVID-19 patients. Of these, lymphopenia has been the most commonly observed disease in COVID-19 [98]. Therefore, it is speculated that the virus might directly infect lymphocytes, which express ACE2 receptors [99]. COVID-19 patients with lymphopenia also seem to have elevated levels of different pro-inflammatory cytokines [100]. Lungs, the primary site for platelet biogenesis, also exhibit a substantial hematopoietic potential [101]. This could be proven in terms of hampered platelet production in the damaged lungs of COVID-19 patients, resulting in thrombocytopenia [102]. Later on, COVID-19-related coagulation abnormalities are often associated with the combination of inflammation, activation of platelets, and endothelial dysfunction [103]. In COVID-19 patients, the higher levels of Factor VIII and von Wille brand factor were reported, which could promote endothelial injury—possibly mediated via ACE2 receptor binding [79,104]. Therefore, the aggravated endothelial injury observed in COVID-19 may lead to a pro-coagulatory state resulting in both macro and microvascular thrombotic episodes. Thus, therapies targeting the restoration and prevention of hematological changes such as endothelial dysfunction and coagulation abnormalities may improve COVID-19 patient outcomes.

The use of EVs for therapeutic and diagnostic purposes in hematological disorders is a emerging field of research. In hematological findings, circulating EVs, particularly those produced by leukocytes, neutrophils, and endothelial cells, have been shown to activate numerous other cells in the blood arteries, including endothelial cells. The intrinsic immunomodulatory characteristics of EVs may also enhance tissue regeneration and vascular repair. Neutrophil-derived EVs autocrinally reduce immune activation and significantly dampen pro-inflammatory cytokine secretion from monocytes [105]. In COVID-19, the major etiologies of ARDS include pneumonia, sepsis, and the invading pathogens [106]. The recruitment of neutrophils to inflamed tissue is required to eliminate pathogens; these neutrophils may secrete EVs at the site of inflammation [55,107] and contribute to reducing cytokine storms caused in COVID-19 [105]. Many studies have shown that endothelial-derived EVs manifest anticoagulant and vasculo-protective potential [108,109] and can aid in plasmin synthesis by plasminogen, which in turn facilitates clot dissolution through amplified fibrinolysis [110]. As a result, we may infer that EVs generated from neutrophils and endothelial cells could be used to treat COVID-19-related coagulation and hematological problems. However, further research is needed to understand the varying roles of EVs produced from various sources in the blood in order to use them as a cell-free treatment for COVID-19 patients with hematologic diseases.

## 3. Translational Potential of EVs in COVID-19 Management

### 3.1. MSC-Derived EVs as Promising Medications

MSCs have been extensively investigated for their therapeutic usefulness in treating various disorders due to their strong regenerative and immunomodulatory capabilities. There are several available sources of MSCs, for instance, bone marrow, adipose tissue, dental pulp, umbilical cord tissue, and amniotic tissue; however, their therapeutic potential may vary depending on their source of origin and the activation of various Toll-like receptors [111,112]. MSCs secrete various cytokines and growth factors such as IL-10, vascular endothelial growth factor (VEGF), hepatocyte growth factor, and keratinocyte growth factor (KGF), which resist fibrosis, mitigate ARDS, and are involved in regeneration and repair of lung damage [113,114]. MSCs could not only restrict aberrant T cell and macrophage production but also enhance their differentiation into functional T cells and anti-inflammatory macrophages, respectively. Additionally, MSCs regulate B cells and dendritic cells, which may be useful in tackling the cytokine storm observed in COVID-19 patients [115,116,117,118]. A plethora of studies have shown that MSC-derived EVs perform similar functions to their parental cells i.e., MSCs, which suggests that the therapeutic efficiency of MSCs in different diseases has been mainly contributed by their secreted EVs [119,120,121,122]. Studies have revealed comparable therapeutic effects of EVs and MSCs in suppressing inflammatory process and edema development in the lungs [123]. Therefore, MSC-derived EVs have gained more attention for exploitation as a cell-free therapy (Figure 2).

MSC-derived EVs are thought to play a therapeutic function in COVID-19 by delivering protective and anti-inflammatory RNAs and proteins to damaged or activated cells in lung tissues [124,125,126]. Reportedly, MSC-EVs are enriched with various types of microRNAs—for instance, let-7, miR-124-3p, miR-21-5p, miR-146a and miR-145 [124,125,127]. Of these, miR-124-3p has been involved in suppressing oxidative stress and inflammatory cytokines by binding to its receptor P2X ligand-gated ion channel 7 (P2X7) [124]. Another miR-21-5p has been associated with reducing lung cell apoptosis through inhibition of PTEN and PDCD4, whilst miR-146a participates in transforming macrophages from pro-inflammatory to anti-inflammatory states by suppressing the NF-κbsignalling pathway [124]. Lastly, miR-145 increases the phagocytic property of macrophages for fast clearance of pathogens at the site of infection [127]. However, our understanding of these EVs is limited, and more studies are required to ensure their robustness and dependability as a viable therapy for combating COVID-19.

### 3.2. Platelet-Derived EV-Based Therapy 

Immunomodulatory properties of convalescent blood products such as whole blood, plasma, and serum aid in wound healing of damaged lungs [128]. In particular, plasma has been successfully used for treatment of COVID-19 patients. During apheresis, many growth factors, neutralizing antibodies, and EVs found in plasma are delivered into patients. EVs in blood circulation are mainly contributed by platelets, which is more than half of the total EVs in the peripheral blood [128,129]. Many studies have demonstrated that plasma-derived EVs express abundant growth factors and participate in the activation of various signaling mechanisms and changes in vascular reactivity, as well as inducing angiogenesis for tissue repair [128,129,130]. Additionally, platelet-derived EVs promote wound healing in several organs by inducing cell proliferation and migration via various signaling pathways [130,131], which suggests that convalescent plasma therapy for COVID-19 patients is mainly contributed to by their circulating EVs. 

Engineered platelet-derived EVs packed with anti-inflammatory molecule TPCA-1 have been shown to be very promising in the curing of pneumonia by inhibiting the inflammatory process and reducing the cytokinestorm in a mouse model [132]. A report has also shown that SARS-CoV-2 binds to ACE2 expressed on endothelial cells and causes damage to endothelial integrity, leading to abnormal angiogenesis [133]. Additionally, it has been proven that platelet-derived EVs enhance the angiogenesis process to repair endothelial integrity after vascular injury [129,130,131,132,133,134]. Another study has also indicated that platelet-EVs carry a variety of growth factors associated with the Akt and Erk pathways, and play key roles in angiogenesis and neurogenesis [135,136,137]. Additionally, the combination of bone marrow stromal cells (BMSCs) and platelet-EVs carrying proteins and non-coding RNAs enhances cell proliferation, migration, and osteogenesis [129,130]. Based on these pieces of evidence, platelet-derived EVs could be deployed as an alternate potential therapeutic option for COVID-19 patients.

### 3.3. EV-Based Vaccines for COVID-19 Prevention

EVs have been characterized as highly stable, less toxic, and low-immunogenic, making them a potential candidate for developing vaccines against COVID-19 [138]. Besides therapeutics, vaccines are very important for preventing SARS-CoV-2 infection in humans. Currently, multiple vaccines are being used worldwide to boost immunity against SARS-CoV-2 over a large population [139]. Several other clinical trials on different vaccines are underway to assess their efficacy and safety against COVID-19. Lipid nanoparticles have been utilized as a vehicle for vaccine development against COVID-19. Vaccines using nanoparticles encapsulated in mRNAs-1273 (BNT162b1, CVnCoV) and saRNAs (LNPnCoVsaRNA) have been employed to prevent COVID-19 virus infection in many countries such as Germany, Belgium, and the United States [140]. Being natural lipid bilayer membrane nano-vesicles, EVs could be an alternate novel avenue in development of vaccines to deal with this pandemic [141,142]. EV-based vaccines carrying SARS-S spike proteins were assessed and compared with adenoviral vector vaccine. Both EV-vaccines and adenoviral vectors have shown encouraging outcomes in neutralizing antibody titers at the same level. After combination with both adenoviral vector and EV-vaccine-carrying S protein, the highest level of neutralization of antibody titer was achieved, which was greater than the convalescent serum of SARS patients [143]. EVs have also been shown to interact with immune cells and activate immune responses to recognize and neutralize specific types of cells [144]. Additionally, EVs have been found to have a higher efficiency than that of soluble proteins utilized in vaccines. This might be attributed to the production of multiple copies of the same viral protein exposed to EVs, which facilitates the cross-linking of EVs and B-cell receptors [145]. These findings imply that EVs containing SARS-CoV-2 components might be used as a COVID-19 vaccine.

### 3.4. Engineered EVs as Delivery Vehicles for COVID-19 Therapy

Exosome therapy can promote endogenous repair and reduce the cytokine storm stimulated by the immune system. It also offer several advantages such as easy storage, low immunogenicity, high stability, and the capability to pass through the BBB [146]. Along with these advantages, their biocompatibility, potentiality for off-shelf availability, and stable membrane composition make them the perfect choice for a drug delivery vehicle [147]. Apart from being endogenous in nature, exosomes can also be engineered and utilized as carriers for delivering specific payloads or drugs. Therefore, the antiviral drugs or immune modulator-loaded exosomes can be delivered directly and internally to targeted sites such as the nasal mucosa and lungs to stimulate antigen-specific immune responses. This strategy of encapsulating drugs into exosomes enhances delivery to targeted organs and minimizes toxicities caused by native drugs. Numerous research works have shown promising outcomes such as successful delivery of therapeutic molecules through EVs. There are two types of advantages observed with engineered EVs: firstly, they cannot be recognized by the host immune system. Secondly, they enhance tissue or cell-specificity for targeted delivery. In order to enhance the targeted delivery and biodistribution of therapeutic components to particular sites in the human body, engineered EVs can be anchored with specific peptides that recognize specific cell surfaces in target tissues [148]. Along with their natural anti-inflammatory effects, these engineered EVs suppress viral replication in host cells, and reduce the cytokine storm and ARDS associated with COVID-19 patients [149].

EVs have been utilized to deliver a variety of therapeutic molecules to treat various lung disorders, including lung inflammation. Small molecules transported by EVs, such as MyD88 siRNA or miR-223/142, have been shown to block the NF-kb signaling pathway or the activation of the Nlrp3 inflammasome in alveolar macrophages, leading to a reduction in lung inflammation [150,151]. While, by using modified surface molecules, EVs may be utilized to target SARS-CoV-2-infected specific cells or tissues for therapeutic purposes [152]. Other compounds, such as nano/antibodies, DNA aptamers, and peptides with caveolin-1 or Ly-6G specificity, were loaded into EVs, allowing anti-inflammatory drugs to be delivered to particular lung epithelial cells and macrophages;this might be a key approach in COVID-19 management to overcome the cytokine storm [153,154]. Another approach using SARS-CoV-2 model cell line (Vero CCL-81 or Vero E6)-derived EVs carrying surface proteins could be utilized for delivering encapsulated drugs to specific alveolar macrophages produced due to SARS-CoV2 infection and reduce the cytokine storm [154]. Hence, these strategies can be very useful in repurposing drugs for treating COVID-19 via EV-based drug delivery.

## 4. Clinical Trials on EVs for COVID-19 Treatments

EVs derived from various sources of MSCs including bone marrow, adipose tissue, peripheral blood, placenta, umbilical cord, amniotic fluid, and gingival tissues are being investigated for development as therapeutics targeting several diseases [155]. We identified nine clinical trial-based studies on EVs for COVID-19 therapy on the clinicaltrials.gov website. Some of these studies are under trial, while two others have been completed and have shown encouraging data in terms of their effectiveness and safety against COVID-19 (Table 1).

In a completed clinical trial (NCT04276987), the efficacy and safety of exosomes derived from adipose tissue-MSCs were assessed in 24 COVID-19 pneumonia patients. In this study, 2 × 10^8^ EVs were administered to the patients, though data have not been published yet. The Direct Biologics company has launched ExoFlo™, an exosome-based drug derived from bone marrow MSCs, and its efficacy has been checked in 24 severely ill COVID-19 patients. A single dose of ExoFlo™ has been found very safe without any severe side effects in those patients, with significant rises in oxygen levels and reduced ARDS symptoms;as additionally, declined levels of acute-phase reactant markers such as C-reactive proteins, ferritin and D-dimers were shown after 14 days of drug administration—although some patients had died during the trial due to other complications not related to ExoFlo^TM^ [156]. The Direct Biologics company has also started a multicentric clinical trial (EXIT-COVID-19) with sixty COVID-19 patients with ARDS and pneumonia to analyze the therapeutic potential of exosomes (NCT04493242). However, recruitment of patients has not been initiated as per the clinicaltrial.gov website. Despite the fact that ExoFloTM has demonstrated a number of therapeutic benefits, several questions are to be answered about its production, including how it was derived from bone marrow-MSCs, its biological activities, infusion dose concentrations, and long-term (72-h) effects after administration to patients [157]. These issues need to addressed prior to its therapeutic use. Additionally, there is a need for more EV-based clinical trials on large numbers of severe COVID-19 patients in order to evaluate their therapeutic relevance in combating this pandemic.

## 5. Challenges in Designing EVs as Therapeutic Candidates

EV-based therapy may serve as a potential approach for the treatment of patients with COVID-19. These exosomes are in the limelight in recent times due to their potential role in therapeutics for different diseases. However, clinical trials are limited in number due to the difficulties associated with these vesicles, which must be addressed in order to develop EVs as a therapeutic alternative.

One of the major existing challenges is the maintenance and functional behavior of the EVs [158]. Exosomes produced from MSCs and other parts of the body have been found to be more stable and viable at −80 °C for longer periods of time. However, cluster formation occurs during freeze–thaw cycles [159]. Storage at low temperatures and transportation of EVs may result in reduced translational activity of the exosomes [160]. Therefore, considering these factors, alternative strategies should be planned for safe handling, maintenance, and transportation of EVs. To reduce or overcome these issues, freeze-dried exosomes have shown promising results for preservation at room temperature. Additionally, this process enhances the shelf life of exosomes and reduces storage facility demands at very low temperatures, as well as reducing transportation costs [158]. However, clustering or aggregation of EVs and debasement of their bioactive components could raise an issue during the freeze-drying process. This may be sorted out with the addition of different stabilizers such as glucose, sucrose, and trehalose, resulting in the formation of a hydration circle surrounding EVs throughout the freeze-drying process, preventing their aggregation and maintaining their membrane integrity.

Another difficulty with modified EVs as drug delivery vehicles is that their deposition in specific cells, tissues, or organs might have a variety of negative consequences, affecting their efficacy and safety in curing illness. To overcome this, coating of EVs with synthetic materials such as polyethylene glycol or streptavidin have been shown to increase vesicle bioavailability and extravasation capacity, and thus could increase their accumulation in lung tissue affected by COVID-19. Engineered EVs with certain specific proteins or peptides have exhibited the ability to increase the tissue specificity of EVs, which might help us better comprehend such customized treatment approaches in the future [148].

One of the most prominent issues with EVs is their source of origin, since the majority of EVs derived from cancer cell cultures include tumorigenic miRNAs, which have been proven to dramatically increase the process of carcinogenesis [161,162]. Hence, they are not appropriate to be utilized as therapeutic agents. Thus, the source of EVs should be considered in order to better fulfil the needs of EV-based therapies. 

Another limitation with EVs is the lack of standardized functional assays to determine the activity of EV preparations [163]. The techniques used in the isolation and purification of EVs for large-scale production have not been well standardized up to the gold standard, making it challenging to employ EVs as a therapeutic alternative [164]. Gel filtration chromatography and ultracentrifugation are being used for the isolation and purification of EVs. Ultracentrifugation, on the other hand, has a number of limitations, including high equipment costs, long run durations, labor-intensiveness, and restricted portability, whereas gel filtration chromatography has low yields and is time-consuming [148,165]. Overall, perfection of these methods to achieve highly purified EV yields at large scales is very challenging. Therefore, genetic engineering can play a crucial role in developing strategies for improved therapeutic function of EVs. However, there are still many issues remaining for debate, such as transformation and differentiation of EV properties.

## 6. Concluding Remarks and Future Directions

To combat the COVID-19 pandemic, a multidirectional approach must be established to reduce its pervasiveness. For the treatment of severe cases and prevention of aggravation, MSCs and exosome therapy could be potential therapeutic options. MSCs have the potential to stimulate endothelium and epithelial healing by transferring EV components across cells via intercellular communication and secretion of soluble factors— resulting in increased alveolar fluid clearance, making them a viable therapeutic option for COVID-19 treatment. Following this, several clinical trials are being conducted to determine the effectiveness and safety of EVs; however, only a few have been accomplished. The symptoms of pneumonia, ARDS, inflammation, and sepsis, which are important contributors to COVID-19 pathogenesis, have demonstrated steady improvement with EV-based treatment. However, the most effective and safest method of EV distribution has yet to be identified. The immunomodulatory, regenerative, and antibacterial properties of EVs have been ascribed as their contribution in COVID-19 therapy. Exosomes also offer multiple benefits, including the capacity to transport drugs, high biocompatibility, minimal immunogenicity, and cell targeting in host cells, making them an attractive choice for off-shelf therapies. Based on the numerous clinical findings stated above, EVs can be established as a cell-free therapy and as drug delivery vehicles in COVID-19 management. However, procedures for isolating EVs, as well as effectiveness and safety measures, and appropriate ethical norms should all be well standardized.

## Figures and Tables

**Table 1:** Clinical trials registered on ClinicalTrials.gov until May 16, 2021, utilizing extracellular vesicles and/or exosomes for the treatment of COVID-19.

Study Identifier	Disease Condition	Source of EVs	Study Phase Study Type Enrolled Patients	Route of Administration	Recruitment Status	Country/Year
NCT04602442	SARS-CoV-2 Pneumonia	MSC	Phase II Interventional 90	Inhalation	Enrolling by invitation	Russia/ 2020
NCT04798716	Coronavirus Infections ARDS	MSCs	Phase I and II Interventional 55	Intravenous	Not yet recruited	USA/ 2021
NCT04747574	SARS-CoV-2	Drug: Exosomes-CD24	Phase Interventional 35	Intravenous	Recruiting	Israel/ 2021
NCT04491240	Covid19 SARS-CoV-2 PNEUMONIA	Drug Exosomes	Phase I and II Interventional 30	Inhalation	Completed	Russia/ 2020
NCT04389385	Corona Virus Infection Pneumonia	T-Cells	Phase I Interventional 60	Intravenous	Recruiting	Turkey/ 2020
NCT04276987	Coronavirus	Adipose tissue MSCs	Phase I Interventional 24	Inhalation	Completed	China/ 2020
NCT04384445	Corona Virus Infection COVID-19 SARS ARDS	Biological Zofin from human Amniotic fluids	Phase I and II Interventional 20	Intravenous	Recruiting	USA/ 2021
NCT04657406	Covid19 Corona Virus Infection SARS ARDS	Drug Zofin ^TM^ from human amniotic fluid (HAF)	Phase –not available Interventional Not available	Intravenous	Available	Not available/ 2020
NCT04493242	Covid19 ARDS Pneumonia Viral	Bone marrow	Phase II 60	Intravenous	No yet recruiting	US / 2021

## Data Availability

This study did not report any data.

## References

[1] Du J., Dong L., Wang T., Yuan C., Fu R., Zhang L., Liu B., Zhang M., Yin Y., Qin J. (2020). Psychological symptoms among frontline healthcare workers during COVID-19 outbreak in Wuhan. Gen. Hosp. Psychiatry.

[2] Huang C., Wang Y., Li X., Ren L., Zhao J., Hu Y., Zhang L., Fan G., Xu J., Gu X. (2020). Clinical Features of Patients Infected with 2019 Novel Coronavirus in Wuhan, China. Lancet.

[3] Nagoor Meeran M.F., Javed H., Sharma C., Goyal S.N., Kumar S., Jha N.K., Ojha S. (2021). Can Echinacea Be a Potential Candidate to Target Immunity, Inflammation, and Infection-The Trinity of Coronavirus Disease 2019. Heliyon.

[4] Zhou F., Yu T., Du R., Fan G., Liu Y., Liu Z., Xiang J., Wang Y., Song B., Gu X. (2020). Clinical Course and Risk Factors for Mortality of Adult Inpatients with COVID-19 in Wuhan, China: A Retrospective Cohort Study. Lancet.

[5] Meeran M.F.N., Sharma C., Goyal S.N., Kumar S., Ojha S. (2021). CB2 Receptor-Selective Agonists as Candidates for Targeting Infection, Inflammation, and Immunity in SARS-CoV-2 Infections. Drug Dev. Res..

[6] Sahin A.R., Erdogan A., Mutlu Agaoglu P., Dineri Y., Cakirci A.Y., Senel M.E., Okyay R.A., Tasdogan A.M. (2020). 2019 Novel Coronavirus (COVID-19) Outbreak: A Review of the Current Literature. EURASIAN J. Med. Oncol..

[7] Kumar A., Prasoon P., Sekhawat P.S., Pareek V., Faiq M.A., Kumari C., Narayan R.K., Kulandhasamy M., Kant K. (2021). Pathogenesis guided therapeutic management of COVID-19: An immunological perspective. Int. Rev. Immunol..

[8] Tsuchiya A., Takeuchi S., Iwasawa T., Kumagai M., Sato T., Motegi S., Ishii Y., Koseki Y., Tomiyoshi K., Natsui K. (2020). Therapeutic potential of mesenchymal stem cells and their exosomes in severe novel coronavirus disease 2019 (COVID-19) cases. Inflamm. Regen..

[9] Al-Khawaga S., Abdelalim E.M. (2020). Potential application of mesenchymal stem cells and their exosomes in lung injury: An emerging therapeutic option for COVID-19 patients. Stem Cell Res. Ther..

[10] Sanyaolu A., Okorie C., Marinkovic A., Patidar R., Younis K., Desai P., Hosein Z., Padda I., Mangat J., Altaf M. (2020). Comorbidity and Its Impact on Patients with COVID-19. SN Compr. Clin. Med..

[11] Pocsfalvi G., Mammadova R., Ramos Juarez A.P., Bokka R., Trepiccione F., Capasso G. (2020). COVID-19 and Extracellular Vesicles: An Intriguing Interplay. Kidney Blood Press. Res..

[12] Fujita Y., Hoshina T., Matsuzaki J., Kadota T., Fujimoto S., Kawamoto H., Watanabe N., Sawaki K., Sakamoto Y., Miyajima M. (2020). Early Prediction of COVID-19 Severity Using Extracellular Vesicles and Extracellular RNAs. medRxiv.

[13] Jabbari N., Karimipour M., Khaksar M., Akbariazar E., Heidarzadeh M., Mojarad B., Aftab H., Rahbarghazi R., Rezaie J. (2020). Tumor-derived extracellular vesicles: Insights into bystander effects of exosomes after irradiation. Lasers Med. Sci..

[14] Kowal J., Tkach M., Théry C. (2014). Biogenesis and Secretion of Exosomes. Curr. Opin. Cell Biol..

[15] Jeppesen D.K., Fenix A.M., Franklin J.L., Higginbotham J.N., Zhang Q., Zimmerman L.J., Liebler D.C., Ping J., Liu Q., Evans R. (2019). Reassessment of Exosome Composition. Cell.

[16] Hurley J.H. (2015). ESCRTs Are Everywhere. EMBO J..

[17] Zhang J., Kumar S., Jayachandran M., Herrera Hernandez L.P., Wang S., Wilson E.M., Lieske J.C. (2021). Excretion of Urine Extracellular Vesicles Bearing Markers of Activated Immune Cells and Calcium/Phosphorus Physiology Differ between Calcium Kidney Stone Formers and Non-Stone Formers. BMC Nephrol..

[18] Jayachandran M., Yuzhakov S.V., Kumar S., Larson N.B., Enders F.T., Milliner D.S., Rule A.D., Lieske J.C. (2020). Specific Populations of Urinary Extracellular Vesicles and Proteins Differentiate Type 1 Primary Hyperoxaluria Patients without and with Nephrocalcinosis or Kidney Stones. Orphanet J. Rare Dis..

[19] Nabhan J.F., Hu R., Oh R.S., Cohen S.N., Lu Q. (2012). Formation and release of arrestin domain-containing protein 1-mediated microvesicles (ARMMs) at plasma membrane by recruitment of TSG101 protein. Proc. Natl. Acad. Sci. USA.

[20] Wang Q., Lu Q. (2017). Plasma Membrane-Derived Extracellular Microvesicles Mediate Non-Canonical Intercellular NOTCH Signaling. Nat. Commun..

[21] Li B., Antonyak M.A., Zhang J., Cerione R.A. (2012). RhoA Triggers a Specific Signaling Pathway That Generates Transforming Microvesicles in Cancer Cells. Oncogene.

[22] Yang J.-M., Gould S.J. (2013). The Cis-Acting Signals That Target Proteins to Exosomes and Microvesicles. Biochem. Soc. Trans..

[23] Xia X., Yuan P., Liu Y., Wang Y., Cao W., Zheng J.C. (2021). Emerging roles of extracellular vesicles in COVID-19, a double-edged sword?. Immunology.

[24] Dong M., Zhang J., Ma X., Tan J., Chen L., Liu S., Xin Y., Zhuang L. (2020). ACE2, TMPRSS2 distribution and extrapulmonary organ injury in patients with COVID-19. Biomed. Pharmacother..

[25] Kumar A., Prasoon P., Kumari C., Pareek V., Faiq M.A., Narayan R.K., Kulandhasamy M., Kant K. (2021). SARS-CoV-2-specific virulence factors in COVID-19. J. Med. Virol..

[26] Bhaskar S., Sinha A., Banach M., Mittoo S., Weissert R., Kass J.S., Rajagopal S., Pai A.R., Kutty S. (2020). Cytokine Storm in COVID-19—Immunopathological Mechanisms, Clinical Considerations, and Therapeutic Approaches: The REPROGRAM Consortium Position Paper. Front. Immunol..

[27] Li Y., Zhou W., Yang L., You R. (2020). Physiological and pathological regulation of ACE2, the SARS-CoV-2 receptor. Pharmacol. Res..

[28] Chatterjee S. (2020). Understanding the Nature of Variations in Structural Sequences Coding for Coronavirus Spike, Envelope, Membrane and Nucleocapsid Proteins of SARS-CoV-2.

[29] Hussain A., Kaler J., Tabrez E., Tabrez S., Tabrez S.S.M. (2020). Novel COVID-19: A Comprehensive Review of Transmission, Manifestation, and Pathogenesis. Cureus.

[30] Amawi H., Abu Deiab G.I., Aljabali A.A.A., Dua K., Tambuwala M.M. (2020). COVID-19 pandemic: An overview of epidemiology, pathogenesis, diagnostics and potential vaccines and therapeutics. Ther. Deliv..

[31] Moll G., Rasmusson-Duprez I., von Bahr L., Connolly-Andersen A.-M., Elgue G., Funke L., Hamad O.A., Lönnies H., Magnusson P.U., Sanchez J. (2012). Are Therapeutic Human Mesenchymal Stromal Cells Compatible with Human Blood?. Stem Cells.

[32] Pacienza N., Lee R.H., Bae E.-H., Kim D.-K., Liu Q., Prockop D.J., Yannarelli G. (2019). In Vitro Macrophage Assay Predicts the In Vivo Anti-inflammatory Potential of Exosomes from Human Mesenchymal Stromal Cells. Mol. Ther. Methods Clin. Dev..

[33] Campagnoli C., Roberts I.A., Kumar S., Bennett P.R., Bellantuono I., Fisk N.M. (2001). Identification of mesenchymal stem/progenitor cells in human first-trimester fetal blood, liver, and bone marrow. Blood.

[34] Neudecker V., Brodsky K.S., Clambey E.T., Schmidt E.P., Packard T.A., Davenport B., Standiford T.J., Weng T., Fletcher A.A., Barthel L. (2017). Neutrophil transfer of miR-223 to lung epithelial cells dampens acute lung injury in mice. Sci. Transl. Med..

[35] Zou X., Chen K., Zou J., Han P., Hao J., Han Z. (2020). Single-cell RNA-seq data analysis on the receptor ACE2 expression reveals the potential risk of different human organs vulnerable to 2019-nCoV infection. Front. Med..

[36] Gheblawi M., Wang K., Viveiros A., Nguyen Q., Zhong J.C., Turner A.J., Raizada M.K., Grant M.B., Oudit G.Y. (2020). Angiotensin-Converting Enzyme 2: SARS-CoV-2 Receptor and Regulator of the Renin-Angiotensin System. Circ. Res..

[37] Fox S.E., Li G., Akmatbekov A., Harbert J.L., Lameira F.S., Brown J.Q., Vander Heide R.S. (2020). Unexpected Features of Cardiac Pathology in COVID-19 Infection. Circulation.

[38] Bradley B.T., Maioli H., Johnston R., Chaudhry I., Fink S.L., Xu H., Najafian B., Deutsch G., Lacy J.M., Williams T. (2020). Histopathology and ultrastructural findings of fatal COVID-19 infections in Washington State: A case series. Lancet.

[39] Wu C., Hu X., Song J., Du C., Xu J., Yang D., Chen D., Zhong M., Jiang J., Xiong W. (2020). Heart injury signs are associated with higher and earlier mortality in coronavirus disease 2019 (COVID-19). medRxiv.

[40] Libby P. (2020). The Heart in COVID-19: Primary Target or Secondary Bystander?. JACC Basic Transl. Sci..

[41] Rapkiewicz A.V., Mai X., Carsons S.E., Pittaluga S., Kleiner D.E., Berger J.S., Thomas S., Adler N.M., Charytan D.M., Gasmi B. (2020). Megakaryocytes and platelet-fibrin thrombi characterize multi-organ thrombosis at autopsy in COVID-19: A case series. EClinicalMedicine.

[42] Long B., Brady W.J., Koyfman A., Gottlieb M. (2020). Cardiovascular complications in COVID-19. Am. J. Emerg. Med..

[43] Wiklander O.P.B., Brennan M.Á., Lötvall J., Breakefield X.O., Andaloussi S.E. (2019). Advances in therapeutic applications of extracellular vesicles. Sci. Transl. Med..

[44] Lai R.C., Arslan F., Lee M.M., Sze N.S.K., Choo A., Chen T.S., Salto-Tellez M., Timmers L., Lee C.N., El Oakley R.M. (2010). Exosome secreted by MSC reduces myocardial ischemia/reperfusion injury. Stem Cell Res..

[45] Arslan F., Lai R.C., Smeets M.B., Akeroyd L., Choo A., Aguor E.N.E., Timmers L., van Rijen H.V., Doevendans P.A., Pasterkamp G. (2013). Mesenchymal stem cell-derived exosomes increase ATP levels, decrease oxidative stress and activate PI3K/Akt pathway to enhance myocardial viability and prevent adverse remodeling after myocardial ischemia/reperfusion injury. Stem Cell Res..

[46] Teng X., Chen L., Chen W., Yang J., Yang Z., Shen Z. (2015). Mesenchymal Stem Cell-Derived Exosomes Improve the Microenvironment of Infarcted Myocardium Contributing to Angiogenesis and Anti-Inflammation. Cell. Physiol. Biochem..

[47] Bian S., Zhang L., Duan L., Wang X., Min Y., Yu H. (2014). Extracellular vesicles derived from human bone marrow mesenchymal stem cells promote angiogenesis in a rat myocardial infarction model. J. Mol. Med..

[48] Feng Y., Huang W., Wani M., Yu X., Ashraf M. (2014). Ischemic Preconditioning Potentiates the Protective Effect of Stem Cells through Secretion of Exosomes by Targeting Mecp2 via MiR-22. PLoS ONE.

[49] Yu B., Kim H.W., Gong M., Wang J., Millard R.W., Wang Y., Ashraf M., Xu M. (2015). Exosomes Secreted from GATA-4 Overexpressing Mesenchymal Stem Cells Serve as a Reservoir of Anti-Apoptotic microRNAs for Cardioprotection. Int. J. Cardiol..

[50] Zhao Y., Sun X., Cao W., Ma J., Sun L., Qian H., Zhu W., Xu W. (2015). Exosomes Derived from Human Umbilical Cord Mesenchymal Stem Cells Relieve Acute Myocardial Ischemic Injury. Stem Cells Int..

[51] Cheng Y., Luo R., Wang K., Zhang M., Wang Z., Dong L., Li J., Yao Y., Ge S., Xu G. (2020). Kidney disease is associated with in-hospital death of patients with COVID-19. Kidney Int..

[52] Naicker S., Yang C.-W., Hwang S.-J., Liu B.-C., Chen J.-H., Jha V. (2020). The Novel Coronavirus 2019 epidemic and kidneys. Kidney Int..

[53] Diao B., Wang C., Wang R., Feng Z., Tan Y., Wang H., Wang C., Liu L., Liu Y., Liu Y. (2021). Human Kidney Is a Target for Novel Severe Acute Respiratory Syndrome Coronavirus 2 Infection. Nat. Commun..

[54] Pitt J.M., Kroemer G., Zitvogel L. (2021). Extracellular Vesicles: Masters of Intercellular Communication and Potential Clinical Interventions. J. Clin. Investig..

[55] Camussi G., Deregibus M.C., Bruno S., Cantaluppi V., Biancone L. (2010). Exosomes/microvesicles as a mechanism of cell-to-cell communication. Kidney Int..

[56] Aghajani Nargesi A., Lerman L.O., Eirin A. (2017). Mesenchymal Stem Cell-Derived Extracellular Vesicles for Kidney Repair: Current Status and Looming Challenges. Stem Cell Res. Ther..

[57] Tsuji K., Kitamura S., Wada J. (2020). Immunomodulatory and Regenerative Effects of Mesenchymal Stem Cell-Derived Extracellular Vesicles in Renal Diseases. Int. J. Mol. Sci..

[58] Lv L., Wu W., Feng Y., Li Z., Tang T., Liu B. (2018). Therapeutic application of extracellular vesicles in kidney disease: Promises and challenges. J. Cell. Mol. Med..

[59] Lv L.L., Tang P.M.-K., Li C.J., You Y.K., Li J., Huang X.-R., Ni J., Feng M., Liu B.C., Lan H.-Y. (2017). The pattern recognition receptor, Mincle, is essential for maintaining the M1 macrophage phenotype in acute renal inflammation. Kidney Int..

[60] Meng X.-M., Tang P.M.-K., Li J., Lan H.Y. (2015). Macrophage Phenotype in Kidney Injury and Repair. Kidney Dis..

[61] Shen B., Liu J., Zhang F., Wang Y., Qin Y., Zhou Z., Qiu J., Fan Y. (2016). CCR2 Positive Exosome Released by Mesenchymal Stem Cells Suppresses Macrophage Functions and Alleviates Ischemia/Reperfusion-Induced Renal Injury. Stem Cells Int..

[62] Phipps M.M., Barraza L.H., LaSota E.D., Sobieszczyk M.E., Pereira M.R., Zheng E.X., Fox A.N., Zucker J., Verna E.C. (2020). Acute Liver Injury in COVID-19: Prevalence and Association with Clinical Outcomes in a Large U.S. Cohort. Hepatology.

[63] Xu Z., Shi L., Wang Y., Zhang J., Huang L., Zhang C., Liu S., Zhao P., Liu H., Zhu L. (2020). Pathological findings of COVID-19 associated with acute respiratory distress syndrome. Lancet Respir. Med..

[64] Chai X., Hu L., Zhang Y., Han W., Lu Z., Ke A., Zhou J., Shi G., Fang N., Fan J. (2020). Specific ACE2 Expression in Cholangiocytes May Cause Liver Damage After 2019-nCoV Infection. biorxiv.

[65] Robinson M.W., Harmon C., O’Farrelly C. (2016). Liver Immunology and Its Role in Inflammation and Homeostasis. Cell Mol. Immunol..

[66] Chand N., Sanyal A.J. (2007). Sepsis-induced cholestasis. Hepatology.

[67] Fix O.K., Hameed B., Fontana R.J., Kwok R.M., McGuire B.M., Mulligan D.C., Pratt D.S., Russo M.W., Schilsky M.L., Verna E.C. (2020). Clinical Best Practice Advice for Hepatology and Liver Transplant Providers During the COVID-19 Pandemic: AASLD Expert Panel Consensus Statement. Hepatology.

[68] Tan C.Y., Lai R.C., Wong W., Dan Y.Y., Lim S.-K., Ho H.K. (2014). Mesenchymal stem cell-derived exosomes promote hepatic regeneration in drug-induced liver injury models. Stem Cell Res. Ther..

[69] Li T., Yan Y., Wang B., Qian H., Zhang X., Shen L., Wang M., Zhou Y., Zhu W., Li W. (2013). Exosomes Derived from Human Umbilical Cord Mesenchymal Stem Cells Alleviate Liver Fibrosis. Stem Cells Dev..

[70] Fiore E., Domínguez L.M., Bayo J., Malvicini M., Atorrasagasti C., Rodriguez M., Cantero M.J., García M., Yannarelli G., Mazzolini G. (2020). Human umbilical cord perivascular cells-derived extracellular vesicles mediate the transfer of IGF-I to the liver and ameliorate hepatic fibrogenesis in mice. Gene Ther..

[71] Ohara M., Ohnishi S., Hosono H., Yamamoto K., Yuyama K., Nakamura H., Fu Q., Maehara O., Suda G., Sakamoto N. (2018). Extracellular Vesicles from Amnion-Derived Mesenchymal Stem Cells Ameliorate Hepatic Inflammation and Fibrosis in Rats. Stem Cells Int..

[72] Lou G., Chen Z., Zheng M., Liu Y. (2017). Mesenchymal stem cell-derived exosomes as a new therapeutic strategy for liver diseases. Exp. Mol. Med..

[73] Herrera M.B., Fonsato V., Gatti S., Deregibus M.C., Sordi A., Cantarella D., Calogero R., Bussolati B., Tetta C., Camussi G. (2010). Human liver stem cell-derived microvesicles accelerate hepatic regeneration in hepatectomized rats. J. Cell. Mol. Med..

[74] Caress J.B., Castoro R.J., Simmons Z., Scelsa S.N., Lewis R.A., Ahlawat A., Narayanaswami P. (2020). COVID-19-associated Guillain-Barré syndrome: The early pandemic experience. Muscle Nerve.

[75] Paniz-Mondolfi A., Bryce C., Grimes Z., Gordon R.E., Reidy J., Lednicky J., Sordillo E.M., Fowkes M. (2020). Central nervous system involvement by severe acute respiratory syndrome coronavirus-2 (SARS-CoV-2). J. Med. Virol..

[76] Lersy F., Benotmane I., Helms J., Collange O., Schenck M., Brisset J.-C., Chammas A., Willaume T., Lefebvre N., Solis M. (2021). Cerebrospinal Fluid Features in Patients With Coronavirus Disease 2019 and Neurological Manifestations: Correlation with Brain Magnetic Resonance Imaging Findings in 58 Patients. J. Infect. Dis..

[77] Matschke J., Lütgehetmann M., Hagel C., Sperhake J.P., Schröder A.S., Edler C., Mushumba H., Fitzek A., Allweiss L., Dandri M. (2020). Neuropathology of patients with COVID-19 in Germany: A post-mortem case series. Lancet Neurol..

[78] Zubair A.S., McAlpine L.S., Gardin T., Farhadian S., Kuruvilla D.E., Spudich S. (2020). Neuropathogenesis and Neurologic Manifestations of the Coronaviruses in the Age of Coronavirus Disease 2019 A Review. JAMA Neurol..

[79] Varga Z., Flammer A.J., Steiger P., Haberecker M., Andermatt R., Zinkernagel A.S., Mehra M.R., Schuepbach R.A., Ruschitzka F., Moch H. (2020). Endothelial cell infection and endotheliitis in COVID-19. Lancet.

[80] Nascimento Conde J., Schutt W.R., Gorbunova E.E., Mackow E.R. (2020). Recombinant ACE2 Expression Is Required for SARS-CoV-2 To Infect Primary Human Endothelial Cells and Induce Inflammatory and Procoagulative Responses. mBio.

[81] Song E., Zhang C., Israelow B., Lu-Culligan A., Prado A.V., Skriabine S., Lu P., Weizman O.-E., Liu F., Dai Y. (2020). Neuroinvasion of SARS-CoV-2 in human and mouse brain. bioRxiv.

[82] Perrin P., Collongues N., Baloglu S., Bedo D., Bassand X., Lavaux T., Gautier-Vargas G., Keller N., Kremer S., Fafi-Kremer S. (2021). Cytokine release syndrome-associated encephalopathy in patients with COVID-19. Eur. J. Neurol..

[83] Pilotto A., Masciocchi S., Volonghi I., De Giuli V., Caprioli F., Mariotto S., Ferrari S., Bozzetti S., Imarisio A., Risi B. (2021). SARS-CoV-2 encephalitis is a cytokine release syndrome: Evidences from cerebrospinal fluid analyses. Clin. Infect. Dis.

[84] Erickson M.A., Wilson M.L., Banks W.A. (2020). In vitro modeling of blood–brain barrier and interface functions in neuroimmune communication. Fluids Barriers CNS.

[85] Hsuchou H., Kastin A.J., Mishra P.K., Pan W. (2012). C-reactive protein increases BBB permeability: Implications for obesity and neuroinflammation. Cell. Physiol. Biochem..

[86] Erickson M.A., Banks W.A. (2018). Neuroimmune Axes of the Blood-Brain Barriers and Blood-Brain Interfaces: Bases for Physiological Regulation, Disease States, and Pharmacological Interventions. Pharmacol. Rev..

[87] Banks W.A., Freed E.O., Wolf K.M., Robinson S.M., Franko M., Kumar V.B. (2001). Transport of human immunodeficiency virus type 1 pseudoviruses across the blood-brain barrier: Role of envelope proteins and adsorptive endocytosis. J. Virol..

[88] Li Y., Li M., Wang M., Zhou Y., Chang J., Xian Y., Wang D., Mao L., Jin H., Hu B. (2020). Acute cerebrovascular disease following COVID-19: A single center, retrospective, observational study. Stroke Vasc. Neurol..

[89] Jin Y., Ji W., Yang H., Chen S., Zhang W., Duan G. (2020). Endothelial activation and dysfunction in COVID-19: From basic mechanisms to potential therapeutic approaches. Signal. Transduct. Target. Ther..

[90] Andjus P., Kosanović M., Milićević K., Gautam M., Vainio S.J., Jagečić D., Kozlova E.N., Pivoriūnas A., Chachques J.-C., Sakaj M. (2020). Extracellular Vesicles as Innovative Tool for Diagnosis, Regeneration and Protection against Neurological Damage. Int. J. Mol. Sci..

[91] Jin Q., Wu P., Zhou X., Qian H., Xu W. (2021). Extracellular Vesicles: Novel Roles in Neurological Disorders. Stem Cells Int..

[92] Huang J.-H., Yin X.-M., Xu Y., Xu C.-C., Lin X., Ye F.-B., Cao Y., Lin F.-Y. (2017). Systemic Administration of Exosomes Released from Mesenchymal Stromal Cells Attenuates Apoptosis, Inflammation, and Promotes Angiogenesis after Spinal Cord Injury in Rats. J. Neurotrauma.

[93] Li C., Jiao G., Wu W., Wang H., Ren S., Zhang L., Zhou H., Liu H., Chen Y. (2019). Exosomes from Bone Marrow Mesenchymal Stem Cells Inhibit Neuronal Apoptosis and Promote Motor Function Recovery via the Wnt/β-catenin Signaling Pathway. Cell Transplant..

[94] Lambert C., Cisternas P., Inestrosa N.C. (2016). Role of Wnt Signaling in Central Nervous System Injury. Mol. Neurobiol..

[95] Xian P., Hei Y., Wang R., Wang T., Yang J., Li J., Di Z., Liu Z., Baskys A., Liu W. (2019). Mesenchymal stem cell-derived exosomes as a nanotherapeutic agent for amelioration of inflammation-induced astrocyte alterations in mice. Theranostics.

[96] Hong S.-B., Yang H., Manaenko A., Lu J., Mei Q., Hu Q. (2019). Potential of Exosomes for the Treatment of Stroke. Cell Transplant..

[97] Li Y., Cheng Q., Hu G., Deng T., Wang Q., Zhou J., Su X. (2018). Extracellular vesicles in mesenchymal stromal cells: A novel therapeutic strategy for stroke. Exp. Ther. Med..

[98] Araya S., Wordofa M., Mamo M.A., Tsegay Y.G., Hordofa A., Negesso A.E., Fasil T., Berhanu B., Begashaw H., Atlaw A. (2021). The Magnitude of Hematological Abnormalities Among COVID-19 Patients in Addis Ababa, Ethiopia. JMDH.

[99] Xu H., Zhong L., Deng J., Peng J., Dan H., Zeng X., Li T., Chen Q. (2020). High expression of ACE2 receptor of 2019-nCoV on the epithelial cells of oral mucosa. Int. J. Oral Sci..

[100] Unsinger J., McDonough J.S., Shultz L.D., Ferguson T.A., Hotchkiss R.S. (2009). Sepsis-induced human lymphocyte apoptosis and cytokine production in “humanized” mice. J. Leukoc. Biol..

[101] Lefrançais E., Ortiz-Muñoz G., Caudrillier A., Mallavia B., Liu F., Sayah D.M., Thornton E.E., Headley M.B., David T., Coughlin S.R. (2017). The lung is a site of platelet biogenesis and a reservoir for hematopoietic progenitors. Nature.

[102] Liu X., Zhang R., He G. (2020). Hematological findings in coronavirus disease 2019: Indications of progression of disease. Ann. Hematol..

[103] (2020). The Lancet Haematology. COVID-19 coagulopathy: An evolving story. Lancet Haematol..

[104] Escher R., Breakey N., Lämmle B. (2020). Severe COVID-19 infection associated with endothelial activation. Thromb. Res..

[105] Gasser O., Schifferli J.A. (2004). Activated polymorphonuclear neutrophils disseminate anti-inflammatory microparticles by ectocytosis. Blood.

[106] Ware L.B., Matthay M.A. (2000). The acute respiratory distress syndrome. N. Engl. J. Med..

[107] Phillipson M., Kubes P. (2011). The neutrophil in vascular inflammation. Nat. Med..

[108] Mostefai H.A., Meziani F., Mastronardi M.L., Agouni A., Heymes C., Sargentini C., Asfar P., Martinez M.C., Andriantsitohaina R. (2008). Circulating microparticles from patients with septic shock exert protective role in vascular function. Am. J. Respir. Crit. Care Med..

[109] Morel O., Toti F., Morel N., Freyssinet J.-M. (2009). Microparticles in endothelial cell and vascular homeostasis: Are they really noxious?. Haematologica.

[110] Lacroix R., Sabatier F., Mialhe A., Basire A., Pannell R., Borghi H., Robert S., Lamy E., Plawinski L., Camoin-Jau L. (2007). Activation of plasminogen into plasmin at the surface of endothelial microparticles: A mechanism that modulates angiogenic properties of endothelial progenitor cells in vitro. Blood.

[111] Gholizadeh-Ghaleh Aziz S., Alipour S., Ranjbarvan P., Azari A., Babaei G., Golchin A. (2021). Critical Roles of TLRs on the Polarization of Mesenchymal Stem Cells for Cell Therapy of Viral Infections: A Notice for COVID-19 Treatment. Comp. Clin. Pathol..

[112] Raicevic G., Najar M., Stamatopoulos B., De Bruyn C., Meuleman N., Bron D., Toungouz M., Lagneaux L. (2011). The source of human mesenchymal stromal cells influences their TLR profile as well as their functional properties. Cell. Immunol..

[113] Cruz T., Rojas M., Burgess J.K., Heijink I.H. (2019). Preclinical Evidence for the Role of Stem/Stromal Cells in Targeting ARDS. Stem Cell-Based Therapy for Lung Disease.

[114] Zanoni M., Cortesi M., Zamagni A., Tesei A. (2019). The Role of Mesenchymal Stem Cells in Radiation-Induced Lung Fibrosis. Int. J. Mol. Sci..

[115] Watanabe Y., Tsuchiya A., Seino S., Kawata Y., Kojima Y., Ikarashi S., Starkey Lewis P.J., Lu W.-Y., Kikuta J., Kawai H. (2019). Mesenchymal Stem Cells and Induced Bone Marrow-Derived Macrophages Synergistically Improve Liver Fibrosis in Mice. Stem Cells Transl. Med..

[116] Kojima Y., Tsuchiya A., Ogawa M., Nojiri S., Takeuchi S., Watanabe T., Nakajima K., Hara Y., Yamashita J., Kikuta J. (2019). Mesenchymal stem cells cultured under hypoxic conditions had a greater therapeutic effect on mice with liver cirrhosis compared to those cultured under normal oxygen conditions. Regen. Ther..

[117] Ikarashi S., Tsuchiya A., Kawata Y., Kojima Y., Watanabe T., Takeuchi S., Igarashi K., Ideta-Otsuka M., Oki K., Takamura M. (2019). Effects of Human Adipose Tissue-Derived and Umbilical Cord Tissue-Derived Mesenchymal Stem Cells in a Dextran Sulfate Sodium-Induced Mouse Model. BioRes. Open Access.

[118] Kawata Y., Tsuchiya A., Seino S., Watanabe Y., Kojima Y., Ikarashi S., Tominaga K., Yokoyama J., Yamagiwa S., Terai S. (2019). Early injection of human adipose tissue-derived mesenchymal stem cell after inflammation ameliorates dextran sulfate sodium-induced colitis in mice through the induction of M2 macrophages and regulatory T cells. Cell Tissue Res..

[119] Yáñez-Mó M., Siljander P.R.-M., Andreu Z., Zavec A.B., Borràs F.E., Buzas E.I., Buzas K., Casal E., Cappello F., Carvalho J. (2015). Biological properties of extracellular vesicles and their physiological functions. J. Extracell. Vesicles.

[120] Kim D., Nishida H., An S.Y., Shetty A.K., Bartosh T.J., Prockop D.J. (2016). Chromatographically isolated CD63+CD81+ extracellular vesicles from mesenchymal stromal cells rescue cognitive impairments after TBI. Proc. Natl. Acad. Sci. USA.

[121] Rani S., Ryan A.E., Griffin M.D., Ritter T. (2015). Mesenchymal Stem Cell-derived Extracellular Vesicles: Toward Cell-free Therapeutic Applications. Mol. Ther..

[122] Keshtkar S., Azarpira N., Ghahremani M.H. (2018). Mesenchymal stem cell-derived extracellular vesicles: Novel frontiers in regenerative medicine. Stem Cell Res. Ther..

[123] Zhu Y., Feng X., Abbott J., Fang X., Hao Q., Monsel A., Qu J., Matthay M.A., Lee J.W. (2014). Human Mesenchymal Stem Cell Microvesicles for Treatment of E.coli Endotoxin-Induced Acute Lung Injury in Mice. Stem Cells.

[124] Xia X., Wang Y., Huang Y., Zhang H., Lu H., Zheng J.C. (2019). Exosomal miRNAs in central nervous system diseases: Biomarkers, pathological mediators, protective factors and therapeutic agents. Prog. Neurobiol..

[125] Ti D., Hao H., Tong C., Liu J., Dong L., Zheng J., Zhao Y., Liu H., Fu X., Han W. (2015). LPS-preconditioned mesenchymal stromal cells modify macrophage polarization for resolution of chronic inflammation via exosome-shuttled let-7b. J. Transl. Med..

[126] Li J.W., Wei L., Han Z., Chen Z. (2019). Mesenchymal Stromal Cells-Derived Exosomes Alleviate Ischemia/Reperfusion Injury in Mouse Lung by Transporting Anti-Apoptotic MiR-21-5p. Eur. J. Pharmacol..

[127] Hao Q., Gudapati V., Monsel A., Park J.H., Hu S., Kato H., Lee J.H., Zhou L., He H., Lee J.W. (2019). Mesenchymal Stem Cell-Derived Extracellular Vesicles Decrease Lung Injury in Mice. J. Immunol..

[128] Iqbal Yatoo M., Hamid Z., Rather I., Nazir Q.U.A., Bhat R.A., Ul Haq A., Magray S.N., Haq Z., Sah R., Tiwari R. (2020). Immunotherapies and Immunomodulatory Approaches in Clinical Trials—A Mini Review. Hum. Vaccin. Immunother..

[129] Tao S.-C., Guo S.-C., Zhang C.-Q. (2017). Platelet-derived Extracellular Vesicles: An Emerging Therapeutic Approach. Int. J. Biol. Sci..

[130] Torreggiani E., Perut F., Roncuzzi L., Zini N., Baglìo S.R., Baldini N. (2014). Exosomes: Novel effectors of human platelet lysate activity. Eur. Cell. Mater..

[131] Guo S.-C., Tao S.-C., Yin W.-J., Qi X., Yuan T., Zhang C.-Q. (2017). Exosomes derived from platelet-rich plasma promote the re-epithelization of chronic cutaneous wounds via activation of YAP in a diabetic rat model. Theranostics.

[132] Ma Q., Fan Q., Xu J., Bai J., Han X., Dong Z., Zhou X., Liu Z., Gu Z., Wang C. (2020). Calming Cytokine Storm in Pneumonia by Targeted Delivery of TPCA-1 Using Platelet-Derived Extracellular Vesicles. Matter.

[133] Yan Y., Zhou W., Wang Y., Guo Q., Zhao F., Zhu Z., Xing Y., Zhang H., Aljofan M., Jarrahi A.M. (2021). The Potential Role of Extracellular Vesicles in COVID-19 Treatment: Opportunity and Challenge. Front Mol. Biosci..

[134] Varon D., Shai E. (2009). Role of Platelet-Derived Microparticles in Angiogenesis and Tumor Progression. Discov. Med..

[135] Hayon Y., Dashevsky O., Shai E., Brill A., Varon D., Leker R.R. (2012). Platelet Microparticles Induce Angiogenesis and Neurogenesis after Cerebral Ischemia. Curr. Neurovasc. Res..

[136] Hayon Y., Dashevsky O., Shai E., Varon D., Leker R.R. (2012). Platelet Microparticles Promote Neural Stem Cell Proliferation, Survival and Differentiation. J. Mol. Neurosci..

[137] Hayon Y., Shai E., Varon D., Leker R.R. (2012). The Role of Platelets and Their Microparticles in Rehabilitation of Ischemic Brain Tissue. CNS Neurol. Disord. Drug Targets.

[138] Sabanovic B., Piva F., Cecati M., Giulietti M. (2021). Promising Extracellular Vesicle-Based Vaccines against Viruses, Including SARS-CoV-2. Biology.

[139] Kaur S.P., Gupta V. (2020). COVID-19 Vaccine: A Comprehensive Status Report. Virus Res..

[140] Jeyanathan M., Afkhami S., Smaill F., Miller M.S., Lichty B.D., Xing Z. (2020). Immunological considerations for COVID-19 vaccine strategies. Nat. Rev. Immunol..

[141] E Pecq J.-B. (2005). Dexosomes as a Therapeutic Cancer Vaccine: From Bench to Bedside. Blood Cells Mol. Dis..

[142] Tan A., De La Peña H., Seifalian A.M. (2010). The Application of Exosomes as a Nanoscale Cancer Vaccine. Int. J. Nanomed..

[143] Kuate S., Cinatl J., Doerr H.W., Überla K. (2007). Exosomal vaccines containing the S protein of the SARS coronavirus induce high levels of neutralizing antibodies. Virology.

[144] Théry C., Ostrowski M., Segura E. (2009). Membrane vesicles as conveyors of immune responses. Nat. Rev. Immunol..

[145] Polak K., Greze N., Lachat M., Merle D., Chiumento S., Bertrand-Gaday C., Trentin B., Mamoun R.Z. (2020). Extracellular Vesicle-Based Vaccine Platform Displaying Native Viral Envelope Proteins Elicits a Robust Anti-SARS-CoV-2 Response in Mice. bioRxiv.

[146] Kowalewski M., Fina D., Słomka A., Raffa G.M., Martucci G., Lo Coco V., De Piero M.E., Ranucci M., Suwalski P., Lorusso R. (2020). COVID-19 and ECMO: The Interplay between Coagulation and Inflammation—A Narrative Review. Crit. Care.

[147] Millar J.E., von Bahr V., Malfertheiner M.V., Ki K.K., Redd M.A., Bartnikowski N., Suen J.Y., McAuley D.F., Fraser J.F. (2019). Administration of mesenchymal stem cells during ECMO results in a rapid decline in oxygenator performance. Thorax.

[148] De Jong B., Barros E.R., Hoenderop J.G.J., Rigalli J.P. (2020). Recent Advances in Extracellular Vesicles as Drug Delivery Systems and Their Potential in Precision Medicine. Pharmaceutics.

[149] Duong N., Curley K., Brown A., Campanelli A., Do M.A., Levy D., Tantry A., Marriott G., Lu B. (2019). Decoy exosomes as a novel biologic reagent to antagonize inflammation. Int. J. Nanomed..

[150] Zhang D., Lee H., Wang X., Groot M., Sharma L., Cruz C.S.D., Jin Y. (2019). A potential role of microvesicle-containing miR-223/142 in lung inflammation. Thorax.

[151] Zhang D., Lee H., Wang X., Rai A., Groot M., Jin Y. (2018). Exosome-Mediated Small RNA Delivery: A Novel Therapeutic Approach for Inflammatory Lung Responses. Mol. Ther..

[152] Fu S., Wang Y., Xia X., Zheng J.C. (2020). Exosome Engineering: Current Progress in Cargo Loading and Targeted Delivery. NanoImpact.

[153] Lee H., Zhang D., Laskin D.L., Jin Y. (2018). Functional Evidence of Pulmonary Extracellular Vesicles in Infectious and Noninfectious Lung Inflammation. J. Immunol..

[154] Harcourt J., Tamin A., Lu X., Kamili S., Sakthivel S.K., Wang L., Murray J., Queen K., Lynch B., Whitaker B. (2020). Isolation and characterization of SARS-CoV-2 from the first US COVID-19 patient. bioRxiv.

[155] Sharma D., Zhao F. (2021). Updates on clinical trials evaluating the regenerative potential of allogenic mesenchymal stem cells in COVID-19. Npj Regen. Med..

[156] Sengupta V., Sengupta S., Lazo A., Woods P., Nolan A., Bremer N. (2020). Exosomes Derived from Bone Marrow Mesenchymal Stem Cells as Treatment for Severe COVID-19. Stem Cells Dev..

[157] Lim S.K., Giebe B., Weiss D.J., Witwer K.W., Rohde E. (2020). Re: Exosomes Derived from Bone Marrow Mesenchymal Stem Cells as Treatment for Severe COVID-19’’ by Sengupta et al. Stem Cells Dev..

[158] Scarfe L., Taylor A., Sharkey J., Harwood R., Barrow M., Comenge J., Beeken L., Astley C., Santeramo I., Hutchinson C. (2018). Non-Invasive Imaging Reveals Conditions That Impact Distribution and Persistence of Cells after in Vivo Administration. Stem Cell Res. Ther..

[159] Gupta S., Krishnakumar V., Sharma Y., Dinda A.K., Mohanty S. (2021). Mesenchymal Stem Cell Derived Exosomes: A Nano Platform for Therapeutics and Drug Delivery in Combating COVID-19. Stem Cell Rev. Rep..

[160] Plava J., Cihova M., Burikova M., Matuskova M., Kucerova L., Miklikova S. (2019). Recent Advances in Understanding Tumor Stroma-Mediated Chemoresistance in Breast Cancer. Mol. Cancer.

[161] Li L., Li C., Wang S., Wang Z., Jiang J., Wang W., Li X., Chen J., Liu K., Li C. (2016). Exosomes Derived from Hypoxic Oral Squamous Cell Carcinoma Cells Deliver miR-21 to Normoxic Cells to Elicit a Prometastatic Phenotype. Cancer Res..

[162] Meng W., He C., Hao Y., Wang L., Li L., Zhu G. (2020). Prospects and Challenges of Extracellular Vesicle-Based Drug Delivery System: Considering Cell Source. Drug Deliv..

[163] Gimona M., Brizzi M.F., Choo A.B.H., Dominici M., Davidson S.M., Grillari J., Hermann D.M., Hill A.F., de Kleijn D., Lai R.C. (2021). Critical considerations for the development of potency tests for therapeutic applications of mesenchymal stromal cell-derived small extracellular vesicles. Cytotherapy.

[164] Bustos M.L., Huleihel L., Kapetanaki M.G., Lino-Cardenas C.L., Mroz L., Ellis B.M., McVerry B.J., Richards T.J., Kaminski N., Cerdenes N. (2014). Aging Mesenchymal Stem Cells Fail to Protect Because of Impaired Migration and Antiinflammatory Response. Am. J. Respir. Crit. Care Med..

[165] Kurian T.K., Banik S., Gopal D., Chakrabarti S., Mazumder N. (2021). Elucidating Methods for Isolation and Quantification of Exosomes: A Review. Mol. Biotechnol..

